# Neurodegeneration and the immune system: lessons from autoimmune encephalitis

**DOI:** 10.1007/s00415-025-13094-0

**Published:** 2025-04-24

**Authors:** Lucia Campetella, Krzysztof Smolik, Antonio Farina, Bastien Joubert, Sergio Muñiz-Castrillo, Virginie Desestret, Jérôme Honnorat

**Affiliations:** 1 French Reference Center for Paraneoplastic Neurological Syndromes and Autoimmune Encephalitis, Hospices Civils de Lyon, 59 Boulevard Pinel, Bron Cedex, 69677 Lyon, France; 2 MeLiS – UCBL-CNRS UMR 5284 – INSERM U1314, Université Claude Bernard Lyon 1, Lyon, France; 3 Department of Biomedical, Metabolic and Neurosciences, University of Modena and Reggio Emilia, Modena, Italy; 4 Department of Neuroscience, Psychology, Pharmacology and Child Health, University of Florence, Florence, Italy; 5 Neurology Department, Centre Hospitalier Lyon Sud, Hospices Civils de Lyon, 69495 Oullins-Pierre-Bénite, France; 6 Neurology Department, Hospital Universitario 12 de Octubre, Instituto de Investigación Sanitaria Hospital 12 de Octubre (imas12), 28041 Madrid, Spain; 7 Neurocognition and Neuro-Ophthalmology Department, Hôpital Pierre Wertheimer, Hospices Civils de Lyon, Lyon, France

**Keywords:** Neurodegeneration, Autoimmune encephalitis, Neural antibodies, Dementia, IgLON5

## Abstract

The spectrum of autoimmune encephalitis (AE) is expanding to atypical clinical presentations that can mimic neurodegenerative disorders. Among the autoantibodies most frequently associated with manifestations mimicking neurodegenerative disorders—such as dementia, parkinsonism, ataxia and motor neuron disease—IgLON5-, LGI1- and CASPR2-antibodies, predominantly of the IgG4 subclass and associated with specific HLA haplotypes, are the most common. Since these forms of autoimmune encephalitis often lack inflammatory findings in cerebrospinal fluid or magnetic resonance imaging, recognizing clinical ‘red flags’ suggestive of an autoimmune etiology is crucial for accurate diagnosis and timely initiation of immunotherapy. Interestingly, in these forms of autoimmune encephalitis, both inflammatory and neurodegenerative disease mechanisms may be involved. The neurodegenerative component may result directly from antibody effects (e.g., tau deposition in IgLON5-antibody disease) or arise through other mechanisms (e.g., seizures or exacerbation of pre-existing pathology). Moreover, neuroinflammation has recently emerged as a key contributor to primary neurodegenerative disorders. For instance, microglial activation promotes tau pathology propagation, as observed in Alzheimer’s disease and other primary neurodegenerative disorders. While the precise mechanisms linking inflammation and neurodegeneration remain to be fully understood, further research into the interplay between autoimmunity and neurodegeneration may enhance our understanding of disease mechanisms and expand therapeutic opportunities in both autoimmune and neurodegenerative neurological disorders.

## Introduction

The term autoimmune encephalitis (AE) encompasses a spectrum of inflammatory disorders of the central nervous system (CNS), usually characterized by the presence of circulating autoantibodies directed against neural or glial antigens. Among them, the autoantibodies directed against cell surface or synaptic antigens are generally considered to retain pathogenic potential, contrary to those targeting intracellular antigens [1]. Patients typically develop memory deficits, altered consciousness, and psychiatric/behavioral changes, together with seizures and/or focal CNS involvement [2], usually with a subacute onset (< 3 months) and a monophasic course, although a slower onset [3–5] and relapses [6, 7] have been variably reported.

The incidence of AE is rising, in parallel with the discovery of novel autoantibodies and the refinement of diagnostic techniques [8]. Accordingly, the spectrum of known AE manifestations has expanded, highlighting how AE patients may present with slow-onset cognitive impairment, movement disorders, and rarely motor neuron disease resembling neurodegenerative diseases. In these patients, the differential diagnosis can prove challenging, as diagnostic examinations such as brain magnetic resonance imaging (MRI) and cerebrospinal fluid (CSF) analysis may yield no signs of inflammation [9–12], especially in elderly AE patients [13]. Additionally, brain MRI may reveal hippocampal atrophy [14, 15], raising a suspicion for neurodegenerative disorders such as Alzheimer’s disease (AD). Furthermore, neurodegenerative mechanisms may contribute to the pathogenesis of AE, as exemplified by immunoglobulin-like cell adhesion molecule 5 (IgLON5)-antibody (Ab) disease, when an early-stage autoimmune process is followed by pathological tau accumulation in neurons during the later phase of the disease [25].

Neurodegeneration is defined as a progressive loss of neurons and their functions, alongside the frequent deposition of intra- and/or extra-neuronal proteinaceous aggregates [16]. A role for multiple non-neuronal cells in the pathophysiology of neurodegenerative disorders has been recently uncovered, with oligodendrocytes, astrocytes, microglia, and lymphocytes gaining the spotlight as initiators and promoters of neuronal dysfunction and death [17]. For instance, recent studies have shed light on inflammation as a central inducer of neurodegeneration in AD [18], Parkinson’s disease [19] and amyotrophic lateral sclerosis (ALS) [20]. Conversely, neurodegeneration has been demonstrated in primarily inflammatory CNS conditions such as multiple sclerosis [21], possibly affecting long-term outcome and cognitive sequelae.

Thus, inflammation and neurodegeneration appear closely intertwined in several neurological disorders. In clinical practice, it is crucial to correctly distinguish AE from neurodegenerative disorders, given that AE is treatable and prompt initiation of immunotherapy may allow substantial recovery, in contrast to neurodegenerative disorders where disease-modifying treatments are lacking. At the same time, research aiming to gain insight into AE mechanisms can help us shed light on the complex pathophysiological pathways linking inflammation to neurodegeneration. In this review, we provide an overview of AE manifestations that resemble neurodegenerative disorders, highlighting clues to suspect an autoimmune etiology, and summarize the current evidence on neurodegenerative mechanisms in AE, as well as the role of inflammation in neurodegenerative disorders.

## Autoimmune encephalitis mimicking neurodegenerative disorders

**Fig. 1:**
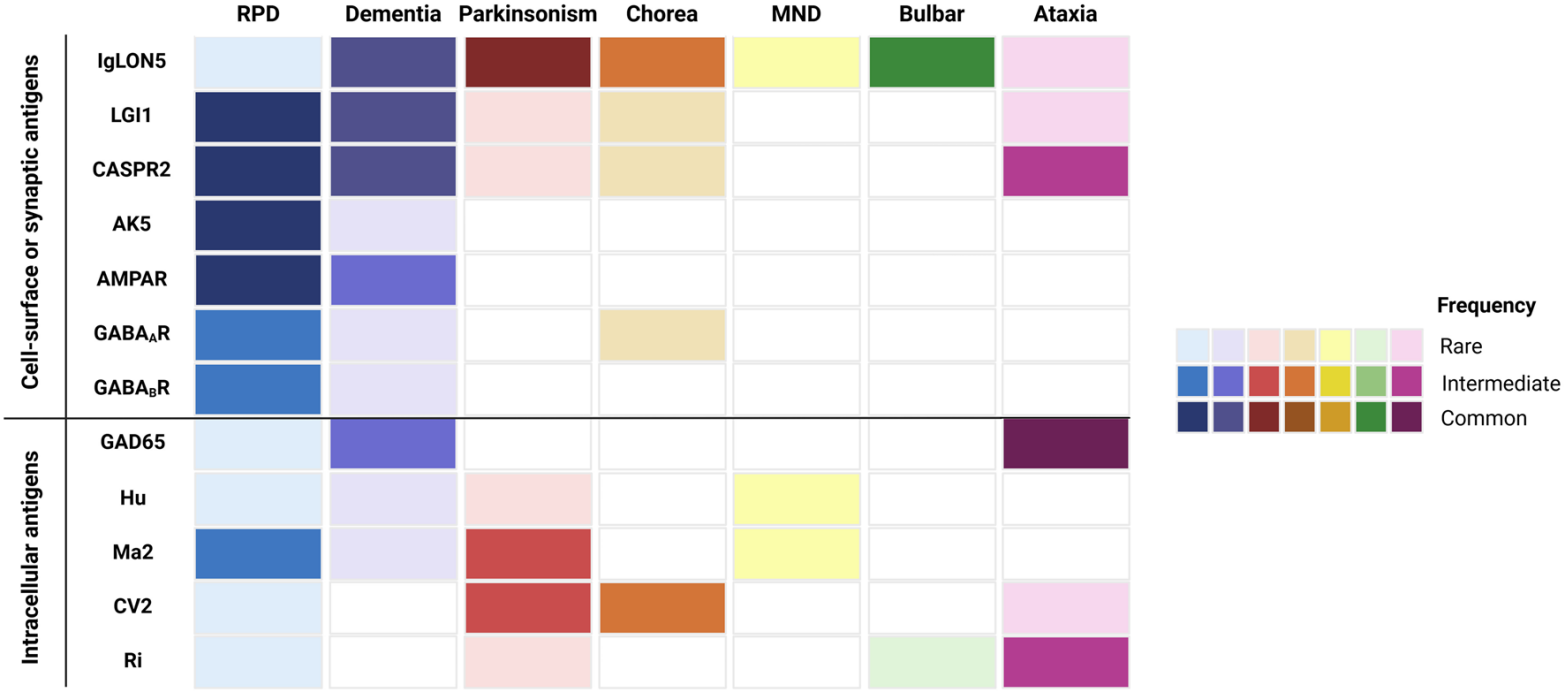
**Clinical manifestations mimicking neurodegenerative disorders in autoimmune encephalitides with antibodies against cell-surface, synaptic or intracellular neural antigens.** Clinical profile and frequency of manifestations resembling neurodegenerative diseases in patients with specific AE subtypes. *MND* motor neuron disease

AEs are classically characterized by cognitive impairment, seizures and psychiatric or behavioral disturbances with a subacute onset, often accompanied by inflammatory CSF findings and areas of T2/FLAIR hyperintensity on brain MRI [2]. Indeed, rapidly evolving limbic encephalitis has long been recognized as the key manifestation of several AE, such as those with leucine-rich glioma-inactivated 1 (LGI1)-, contactin-associated protein-like 2 (CASPR2)-, α-amino-3-hydroxy-5-methyl-4-isoxazolepropionic acid receptor (AMPAR)- and γ-aminobutyric acid B receptor (GABA_B_R)-Abs, although other signs and symptoms may coexist (e.g., neuromyotonia in CASPR2-Ab AE, as highlighted in Table 1). Nonetheless, as time passes, larger cohorts of patients are being collected and investigated, and it is becoming increasingly evident that AE can also manifest with insidious onset and slowly progressive symptoms such as dementia, ataxia, parkinsonism, chorea or muscle weakness [1, 2]. For instance, in a large AE cohort more than one-third of patients with LGI1-, CASPR2-, N-methyl-D-aspartate receptor (NMDAR)- or GABA_B_R-Abs fulfilled diagnostic criteria for dementia [22]. On a similar note, a comprehensive analysis of the disease course of CASPR2-Ab AE revealed that median time from for first symptom to clinical peak was over one year, suggesting that a slowly progressive course is especially common in these patients [3]. Isolated memory impairment has been reported in AMPAR- [15] and LGI1-Ab AE [23], with the consequent risk of misdiagnosis with AD; additionally, patients with adenylate kinase 5 (AK5)-Abs manifest prominent memory deficits, more often with a rapid evolution, although sometimes few patients can be seen only after the inflammatory phase with isolated bi-hippocampal atrophy [24]. Parkinsonism has been described in patients with IgLON5- [25], LGI1- [10,26], Ma2- [27] and Ri-Abs [28], while chorea can be present in AE associated with several autoantibodies targeting both cell surface and intracellular antigens, as illustrated in Table 1 and Fig. 1. Recent studies have also reported AE patients with motor neuron involvement resembling ALS [29–31], further adding to the complex and heterogeneous clinical presentations of AE.

**Table 1:** Diagnostic clues for suspecting autoimmune etiology in neural antibodies-associated syndromes mimicking neurodegenerative disorders

Presenting syndrome	Main presentation	Clinical clues for autoimmune etiology	Neuronal antigen	Paraclinical findings and tumor association
Cognitive impairment	RPD	Anosmia and/or dysgeusia	AK5	MRI (MTL hyperintensity) and CSF (inflammatory) almost always abnormal
		New-onset headache		
		Prominent memory impairment, prosopagnosia		
	RPD/cognitive impairment/dementia	Temporal lobe seizures, status epilepticus	AMPAR	Tumor (SCLC) frequent, MRI often abnormal (MTL hyperintensity), CSF often inflammatory
	RPD/cognitive impairment/dementia	Neuromyotonia	CASPR2	Frequent malignant thymoma if associated with Morvan’s syndrome, MRI often abnormal (MTL hyperintensity), CSF often normal
		Neuropathic pain		
		Dysautonomia		
	Cognitive impairment/dementia	Dysautonomia	CV2/CRMP5	Frequent SCLC/thymoma, MRI often abnormal (multifocal), CSF often inflammatory
		Peripheral neuropathy		
		Myelopathy		
		Ocular manifestations (optic neuritis, retinopathy)		
	RPD	Gastrointestinal symptoms	DPPX	Tumor rare, MRI and CSF often normal
		Weight loss		
		CNS hyperexcitability		
	RPD/cognitive impairment/dementia	Prominent seizures, status epilepticus	GABA_A_R	Tumor rare, MRI (multifocal T2 hyperintensities) and CSF often inflammatory
		Stereotypies		
	RPD	Prominent seizures and status epilepticus	GABA_B_R	Tumor (SCLC) frequent, MRI often abnormal (MTL hyperintensity), CSF often inflammatory
	Cognitive impairment/dementia	Temporal lobe seizures	GAD65	Tumor rare, MRI rarely abnormal, CSF often abnormal (OCBs)
		Psychiatric symptoms		
		Autoimmune comorbidities		
		Cerebellar ataxia		
	RPD	PNS involvement	Hu	Frequent SCLC, MRI often normal, CSF often inflammatory
		Dysautonomia		
	Cognitive impairment/dementia	Sleep disorders (REM/NREM parasomnias, sleep apnea, stridor, RBD)	IgLON5	Tumor rare, MRI often normal, CSF occasionally abnormal (protein elevation)
		Bulbar symptoms		
	RPD/cognitive impairment/dementia	Hyponatremia	LGI1	Tumor rare, MRI often abnormal (MTL hyperintensity), CSF often normal
		Temporal lobe seizures		
		FBDS		
	RPD	Hearing loss	Ma2	Frequent testicular cancer, MRI (diencephalon) and CSF (inflammatory) often abnormal
		Hypersomnia and/or hyperphagia		
		Behavioral changes		
	RPD	Neuropsychiatric manifestations	NMDAR	Frequent teratoma, MRI often normal, CSF often inflammatory
		Chorea and dyskinesia		
		Altered consciousness and hypoventilation		
		Dysautonomia		
		Seizures and/or status epilepticus		
Movement disorders	Ataxia Parkinsonism	EA/EAD responding well to CBZ	CASPR2	Frequent malignant thymoma if associated with Morvan’s syndrome, MRI often abnormal (MTL hyperintensity), CSF often normal
		Neuromyotonia		
		Neuropathic pain		
	Chorea Ataxia	Peripheral neuropathy	CV2/CRMP5	Frequent SCLC/thymoma, MRI often abnormal (multifocal), CSF often inflammatory
		Myelopathy		
		Ocular manifestations (optic neuritis, retinopathy)		
	Ataxia	Temporal lobe seizures	GAD65	Tumor rare, MRI rarely abnormal, CSF often abnormal (OCBs)
		Psychiatric symptoms in young women		
		Autoimmune comorbidities		
	Chorea, dyskinesia (often facial) Parkinsonism Ataxia Dystonia	No response to levodopa	IgLON5	Tumor rare, MRI often normal, CSF occasionally abnormal (protein elevation)
		Sleep disorders (REM/NREM parasomnias, sleep apnea, stridor, RBD)		
		Vocal cord paresis		
	Chorea Ataxia Dyskinesia Myoclonus	PNS involvement	Hu	Frequent SCLC, MRI often normal, CSF often inflammatory
	Chorea and dyskinesia Parkinsonism	Rapidly progressive cognitive impairment	LGI1	Tumor rare, MRI often abnormal (MTL hyperintensity), CSF often normal
		Hyponatremia		
		Temporal lobe seizures		
		FBDS		
	Dystonia Parkinsonism	No response to levodopa	Ma2	Frequent testicular/lung cancer, MRI (diencephalon) and CSF (inflammatory) often abnormal
		Limbic, brainstem or diencephalic involvement		
	Chorea and dyskinesia	Neuropsychiatric symptoms	NMDAR	Frequent teratoma, MRI often normal, CSF often inflammatory
		Young age of onset and female sex		
		Altered consciousness and hypoventilation		
		Dysautonomia		
		Seizures		
	Ataxia Parkinsonism	Multiple movement disorders (myoclonus, tremor, dystonia)	Ri	Frequent SCLC and breast tumors, MRI rarely abnormal, CSF often inflammatory (OCBs)
Motor neuron disease	Motor neuropathy, weakness, fasciculations	Dysautonomia	Hu	Frequent SCLC, MRI often normal, CSF often inflammatory
	Bulbar syndrome, fasciculations	Stridor and/or vocal cord paresis	IgLON5	Tumor rare, MRI often normal, CSF occasionally abnormal (protein elevation)
		Sleep disorders (REM/NREM parasomnias, sleep apnea, stridor, RBD)		
	Bulbar syndrome, fasciculations, weakness	Hypersomnia and/or hyperphagia	Ma2	Frequent testicular/lung cancer, MRI (diencephalon) and CSF (inflammatory) often abnormal
		Hearing loss, vertigo		
		Behavioral changes		

*AK5* adenylate kinase 5, *AMPAR* α-amino-3-hydroxy-5-methyl-4-isoxazolepropionic acid receptor, *CASPR2* contactin-associated protein-like 2, *CBZ* carbamazepine, *CNS* central nervous system, *CRMP5* collapsin response mediator protein 5, *CSF* cerebrospinal fluid, *DPPX* dipeptidyl-peptidase-like protein-6, *EA* episodic ataxia, *EAD* episodic ataxia and dysarthria, *FBDS* faciobrachial dystonic seizures, *GABA*_*A*_*R/GABA*_*B*_*R* γ-aminobutyric acid A/B receptor, *GAD65* glutamic acid decarboxylase 65, *IgLON5* immunoglobulin-like cell adhesion molecule 5, *LGI1* leucine-rich glioma-inactivated 1, *MTL* mesiotemporal lobe, *MRI* magnetic resonance imaging, *NMDAR* N-methyl-D-aspartate receptor, *OCBs* oligoclonal bands, *PNS* peripheral nervous system, *RBD* REM sleep behavior disorder, *REM* rapid eye movement, *RPD* rapidly progressive dementia, *SCLC* small cell lung cancer

In these instances, AE may closely resemble neurodegenerative diseases, and when CSF and MRI findings are unremarkable the differential diagnosis can prove challenging. A comprehensive review found that CSF findings were mostly normal in patients with CASPR2-, LGI1-, GABA_A_R- or glycine receptor-Abs, while those with IgLON5-Abs showed hyperproteinorrachia [9], which could however be justified by the older age of patients [32]. In a cohort of 22 AMPAR-Ab patients CSF pleocytosis was absent in more than half; moreover, four had normal MRI findings [33]. Similarly, MRI in AK5-Ab AE may be normal in 10% of cases [24], and while multifocal T2 hyperintensities are present in most patients with GABA_A_R-Abs [34], MRI is unremarkable in up to 40% of GABA_B_R-Ab AE [35, 36]. Additionally, brain MRI can reveal hippocampal atrophy in several AE [37], especially in LGI1-Ab patients both early in the disease course [12, 38] and in almost all patients during follow-up [14]. Similarly, focal or generalized brain atrophy has also been reported in brain MRIs of patients with CASPR2- [10, 39, 40], GABA_A_R- [41] and GABA_B_R-Abs [42–44], underlining the difficulties that can be encountered in the differential diagnosis with neurodegenerative disorders.

In light of all these findings, when managing patients with signs and symptoms resembling neurodegenerative disorders, recognizing atypical disease patterns and clinical ‘red flags’ suggestive of an autoimmune etiology becomes even more essential, as neural antibody testing is crucial for an accurate diagnosis and the timely initiation of immunotherapy. In the present article, “cognitive impairment” is generally employed to describe any decline in memory or other cognitive skills, while “dementia” is defined as a slow-onset (> 3 months) and gradually progressive cognitive dysfunction impairing daily functioning. Similarly, rapidly progressive dementia (RPD) is defined as a cognitive dysfunction with fast progression leading to dementia within weeks/months [45]. In the following section, we outline the AE which most commonly mimic neurodegenerative disorders due to their clinical presentations, and highlight clues to suspect an autoimmune etiology, as summarized in Fig. 1 and Table 1.

### IgLON5-antibody disease

IgLON5-Ab disease, first described in 2014, is associated with autoantibodies targeting IgLON5, a neuronal cell adhesion protein whose function is still not fully known [46] and the clinical profile is distinct from other diseases associated with neural autoantibodies targeting surface antigens [46]. The clinical course of IgLON5-Ab disease is often slowly progressive with a long diagnostic delay (median of 19–31 months) [4, 47–50]. Even though the most prominent features which initially led to the identification of the disease are sleep disorders (obstructive apneas and/or parasomnias) [46], at diagnosis around 80% of patients present a generalized phenotype [49], combining a wide range of neurological symptoms (including bulbar symptoms, gait difficulties, movement disorders, cognitive impairment). Brain MRI in IgLON5-Ab patients is normal in the majority of cases (74–88%) [25, 48] and the most common alterations are brainstem atrophy, cortical lesions and cerebellar atrophy [47]. The prevalence of CSF pleocytosis is relatively low (less than one third) and, if present, it is usually mild [48, 49, 51].

Up to 90% of patients with IgLON5-Ab disease manifest bulbar symptoms, including dysphagia, dysarthria, and central hypoventilation and/or vocal cord palsy, often leading to respiratory failure [4, 51]. The association of bulbar symptoms with fasciculations (reported in around 10–20%) and/or muscular atrophy or weakness [29, 49, 51, 52] can mimic ALS. However, the presence of parasomnias, vocal cord dysfunction or hyperkinetic movements in patients with motor neuron-like phenotype is atypical for ALS and should prompt antibody testing [29]. Similarly, given that vocal cord palsy can manifest as stridor in about 50% of cases [4], these patients can be misdiagnosed as multiple system atrophy (MSA), especially when other symptoms such as parkinsonism, cerebellar ataxia, severe orthostatic hypotension and sleep parasomnia are present. However, in the presence of the abovementioned symptoms, additional features such as myorhythmia, horizontal eye movement restriction, fasciculations, and painful muscle cramps are atypical for MSA and represent a red flag for alternative diagnoses [53].

Gait impairment, consisting of disequilibrium with postural instability, freezing of gait or parkinsonian gait, and often associated with falls, is frequent (about 70% of patients) [25]. Therefore, in patients with both gait instability and gaze palsy, the clinical presentation may mimic progressive supranuclear palsy (PSP) [25]. However, in IgLON5-Ab disease, as opposed to PSP, the downward gaze limitation is mild or not predominant over upward gaze palsy, and square wave jerks are typically absent [25, 54]. Cognitive impairment and psychiatric symptoms were described in a significant proportion of patients (21–28%) [49, 55] and up to half of patients fulfill criteria for dementia [56]. In cases with an insidious and progressive disease course this may lead to a misdiagnosis of a neurodegenerative condition, such as Lewy body dementia [4, 22, 57] or AD, although in IgLON5-Ab disease the cognitive dysfunction is usually multi-domain and different from the prominent memory impairment typical of AD [58]. Similarly, the patients with both cognitive impairment and chorea (present in up to one third of patients) may be initially diagnosed with Huntington’s disease [25, 51, 59]. However, focal forms of chorea (craniofacial dyskinesias) or early generalized chorea are present in about 30% of patients [25, 60], suggesting an alternative diagnosis and representing a clue for IgLON5-Ab disease.

### LGI1-antibody encephalitis

LGI1-Ab encephalitis is increasingly being recognized as the most common form of AE in adults [61] and typically affects elderly men with a median age at onset of 65–66 years [10, 62]. The typical clinical picture is that of limbic encephalitis, with subacute onset of memory impairment, behavioral changes and seizures, [10, 12] alongside the pathognomonic faciobrachial dystonic seizures (FBDS), namely brief tonic contractions of the arm and/or face (more rarely lower limbs) lasting a few seconds and occurring multiple times a day [63]. The most common MRI finding is T2/FLAIR hyperintensity of mesiotemporal lobe structures [10, 12, 23, 64], although 10–30% of patients have a normal initial brain MRI [23, 64]. Interestingly, hippocampal atrophy can be detected in the acute phase in up to 40% of patients [12, 38], increasing the risk of misdiagnosis with neurodegenerative disorders such as AD. Moreover, CSF findings in LGI1-Ab AE are often uninformative, with inflammatory abnormalities detected in only about one-third of the patients [12, 65, 66] and pleocytosis or oligoclonal bands (OCBs) occurring in less than 20% of cases [23, 66, 67]. Interestingly, some studies found that a significant proportion of LGI1-Ab patients had either a CSF profile compatible with AD [22] or abnormalities in at least one core AD biomarker among total and phosphorylated tau (p-tau), and amyloid beta (Aβ)1–42 [68].

Cognitive dysfunction is the most frequent and usually predominant symptom of LGI1-Ab AE [10, 12, 23, 62], and commonly develops sub-acutely over weeks; thus, LGI1-Ab AE should be considered among the differential diagnoses of rapidly progressive dementia (RPD) [69]. Nonetheless, some LGI1-Ab patients have a slower disease onset, characterized by subtle cognitive and behavioral changes progressing gradually over months, overlapping with neurodegenerative dementias [4, 5, 23]. Among 67 AE patients fulfilling diagnostic criteria for dementia, as previously mentioned, the majority (48%) harbored LGI1-Abs [22]. Proposed clues for an autoimmune etiology in these patients included fluctuating course, myoclonus, or subtle epileptic seizures (e.g., FBDS or nonmotor focal seizures), which are atypical for neurodegenerative disorders [22, 70].

Although cognitive impairment, seizures and behavioral abnormalities typically dominate the clinical picture, some LGI1-Ab patients develop parkinsonism (2–6%) [10, 26] or involuntary movements (2–12%) such as chorea or myoclonus [10, 12, 26, 71], which may raise the suspicion of a neurodegenerative disorder. Notably, a percentage of patients classified as having myoclonus in earlier cohort studies might be explained by FBDS [72]. Nonetheless, several case reports and case series accurately illustrate LGI1-Ab patients with parkinsonism [73–76], chorea [77, 78], and myoclonus [74, 76], reporting misdiagnosis as MSA or PSP [75] and even an initial suspicion of Huntington’s disease [78]. In these patients, subacute onset, rapid evolution of symptoms, early severe cognitive impairment, and seizures represent red flags for neurodegenerative disorders and should prompt alternative diagnoses such as LGI1-Ab AE.

### CASPR2-antibody encephalitis

Autoantibodies targeting CASPR2, a neuronal cell-adhesion protein, are linked to a spectrum of overlapping neurological disorders such as autoimmune limbic encephalitis, Isaacs syndrome (peripheral nerve hyperexcitability with cramps and fasciculations, neurogenic pain, dysautonomia) and Morvan syndrome (peripheral nerve hyperexcitability, severe insomnia, dysautonomia, dream-like enactment behavior, and visual hallucinations) [79]. Inflammatory CSF findings such as a pleocytosis/elevated protein/OCBs are found in about 40% of patients, while MRI is abnormal in up to 50% of patients [3, 80].

Diagnosing CASPR2-Ab AE is challenging due to its phenotypic complexity, its predominance in older male patients (median age at onset of 65 years), and its insidious onset, which can mimic neurodegenerative disorders, particularly when the clinical course is slowly progressive [3, 80]. At disease onset, cognitive impairment and cerebellar ataxia are present in about 10% of patients, reaching up to 94% and 52%, respectively at disease peak [3]. Notably, some patients may present tremor or parkinsonism mimicking purely neurodegenerative movement disorders [80], especially if accompanied by symptoms such as insomnia, dysautonomia, mood disorders, and weight loss [3, 11].

### Paraneoplastic encephalitis associated with autoantibodies targeting intracellular antigens

Paraneoplastic neurological syndromes (PNS) associated with antibodies targeting intracellular neural antigens usually have a subacute presentation with multifocal central and/or peripheral nervous system involvement. The cancer association typically varies according to the autoantibody, e.g., Ri-Abs with breast and lung cancer [81], Ma2-Abs with testicular or lung cancer [82, 83], CV2/CRMP5- [84, 85] and Hu-Abs with small cell lung cancer [30]. CSF analysis shows signs of inflammation in approximately 90% of patients [86], while MRI abnormalities differ in relation to the autoantibody (e.g., 70% of Ma2-Ab and 50% of Hu-Ab patients) [30, 82].

PNS mimicking neurodegenerative disorders are not uncommon. For instance, Ri-Ab AE may manifest with cerebellar ataxia, brainstem dysfunction and/or parkinsonism; moreover, disease onset is sometimes slowly progressive, with 30% of patients reaching a disease plateau in more than 3 months [28], leading to a possible misdiagnosis of PSP or MSA [28, 87]. Chorea, parkinsonism, encephalopathy, and cerebellar involvement are well-documented manifestations in patients harboring CV2/CRMP5-Abs [84, 85]. The signs and symptoms in patients with Ma2-Abs include ALS-, MSA- or PSP-like manifestations [27, 31, 88], with a slower disease onset in a small but significant proportion of patients [30]. Interestingly, Hu-Ab patients may rarely present with a motor neuron-like phenotype (6% in the French Hu-Ab cohort) [30] or parkinsonism [89, 90], sometimes with a progressive disease onset [130], complicating the differential diagnosis with neurodegenerative disorders.

## Neurodegenerative mechanisms in autoimmune encephalitis

**Fig. 2:**
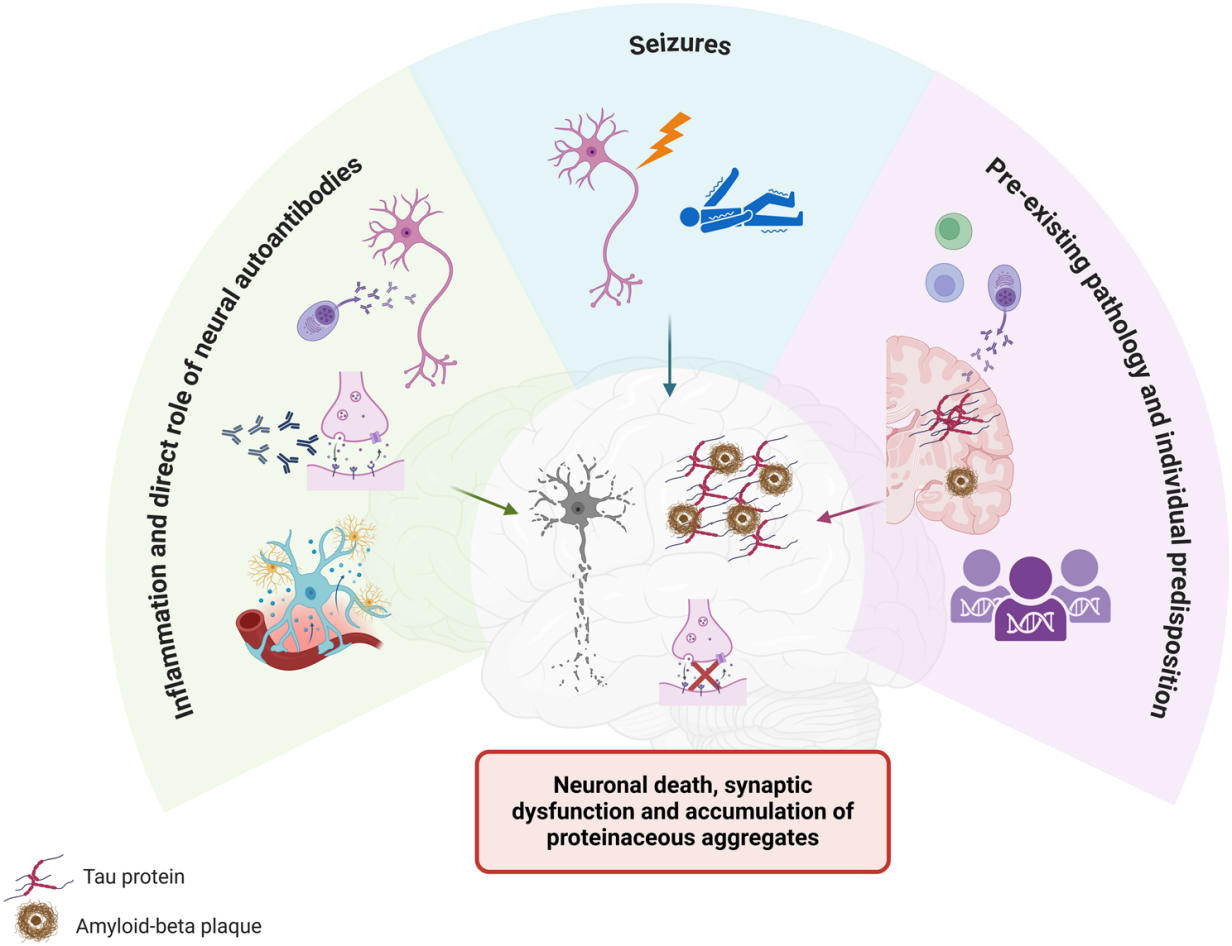
**Proposed mechanisms of neurodegeneration in autoimmune encephalitis.** Inflammation and pathogenic neural autoantibodies (left green panel), seizures (middle blue panel) and pre-existing pathology alongside individual predisposition (right pink panel) through different genetic, molecular and cellular pathways, may all play a role in neurodegeneration leading to neuronal death, synaptic dysfunction and accumulation of proteinaceous aggregates (amyloid, tau) in autoimmune encephalitis patients

The inflammatory pathways in AE have been extensively studied, with the goal of investigating the physiological and pathogenic role of specific autoantibodies, exploring their role in clinical AE manifestations and developing targeted immunomodulatory treatments. Nonetheless, the contribution of neurodegeneration to AE pathophysiology remains underexplored. A deeper understanding of these mechanisms could not only enhance our knowledge of AE but also provide valuable insights into neurodegenerative disorders, potentially revealing novel therapeutic strategies. In this section, we summarize current evidence on neurodegenerative mechanisms in AE, as depicted in Fig. 2, with a particular focus on the role of previously described autoantibodies.

### Inflammation as a trigger for neurodegeneration

The pathogenic effects of AE autoantibodies directed against cell surface or synaptic antigens often result from disruption of the function of their target, or of its interaction with binding partners. The first mechanism has been extensively investigated in NMDAR-Ab AE, where the autoantibodies induce cross-linking and internalization of NMDARs, thus reducing membrane surface expression and impairing synaptic transmission [91–93]. Notably, removal of the autoantibodies restores NMDAR levels and reverses behavioral and memory deficits in mice exposed to NMDAR-Abs [93]. An additional example involves autoantibodies targeting other neurotransmitter receptors (e.g., GABA_A_R and glycine receptors), which can cause impairment in ionotropic transmission in addition to cross-linking-independent receptor internalization [94, 95].

The second mechanism has been well-described especially in AE associated with autoantibodies of the IgG4 subclass, such as LGI1- and CASPR2-Abs [96]. LGI1-Abs target the trans-synaptic complex that LGI1 forms with A Disintegrin And Metalloprotease (ADAM) proteins, leading to altered expression of pre-synaptic Kv1.1 channels and post-synaptic AMPA receptors [96–98]. This results in increased neuronal excitability and impaired synaptic long-term potentiation effects especially in hippocampal neurons [96, 97, 99], consistent with the clinical syndrome of predominant cognitive impairment and seizures. Similarly, CASPR2-Abs impair the interaction between CASPR2 and transient axonal glycoprotein 1 (TAG1), disrupting the recruitment of voltage-gated potassium channels around the nodes of Ranvier in myelinated axons [100, 101]. Additionally, CASPR2-Abs can reduce the surface expression of the glutamatergic receptor GluA1, whose trafficking is regulated by CASPR2 [102, 103], and have been demonstrated to trigger complement-dependent and antibody-dependent cell toxicity in vitro [104]. Although the pathogenic effects of CASPR2-Abs seem mostly reversible, sustained exposure involving complement activation and T- and/or natural killer cell-mediated cytotoxicity might ultimately lead to neuronal death. This damage could be particularly pronounced in the hippocampus, where CASPR2 is abundantly expressed [105], resulting in long-term memory deficits.

In contrast to AE associated with neuronal surface antigens, the autoantibodies targeting intracellular antigens (e.g., AK5-Abs) likely do not play a direct pathogenic role, as neuronal dysfunction and death are primarily mediated by T lymphocytes and macrophages [24].

**Fig. 3:**
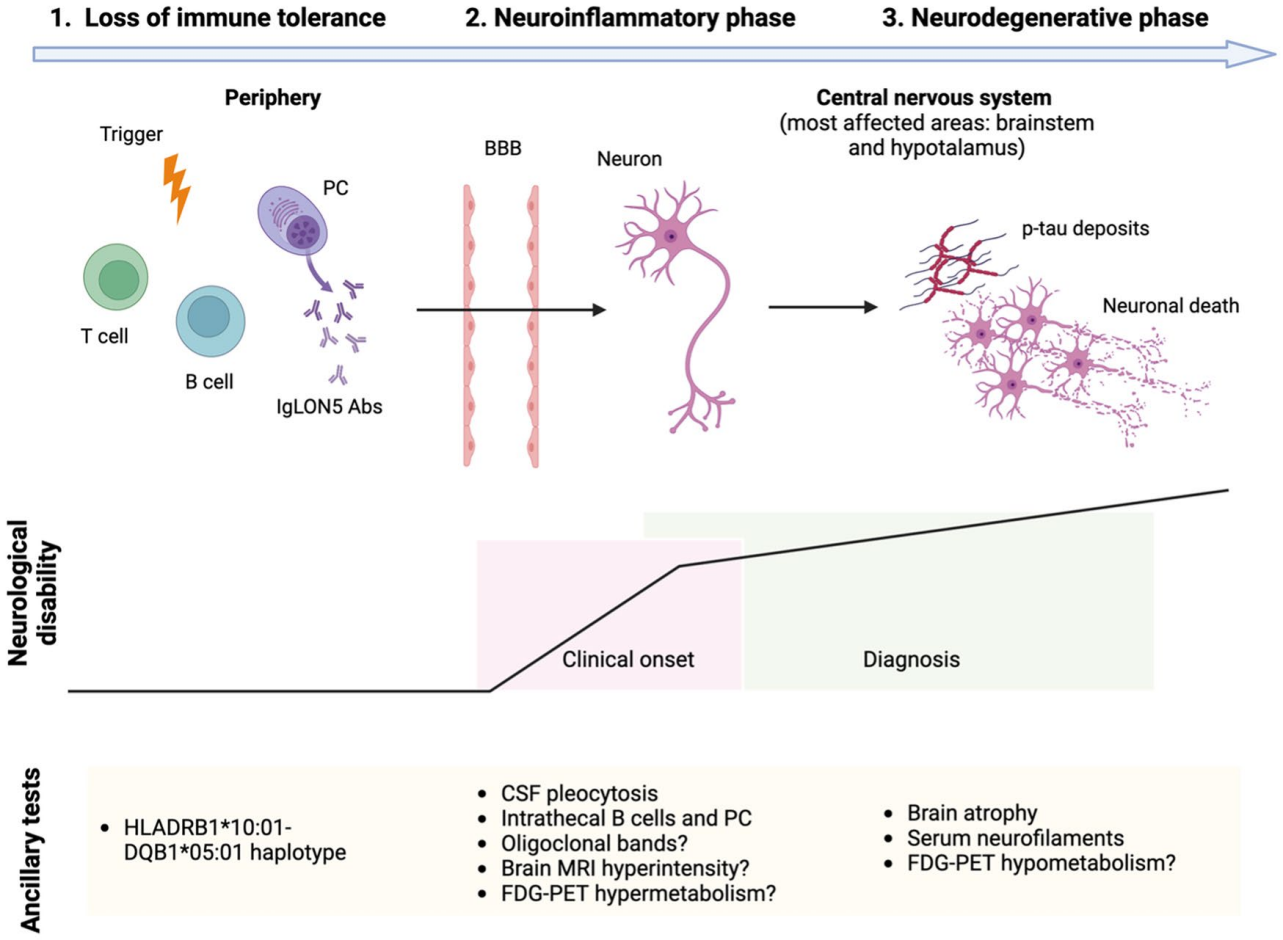
**Proposed immunopathological cascade of IgLON5-Ab disease and its correlation with neurological disability and ancillary tests results.** 1) In predisposed individuals (e.g., HLA-DQB1*05:01 carriers), unknown factors determine the loss of immune tolerance against IgLON5. 2) In the early stage, inflammatory mechanisms, especially humoral, are deemed to prevail; IgG4 antibodies prevent IgLON5 interactions with its binding proteins, resulting in in synaptic neuronal dysfunction. Clinical symptoms may appear in this phase, but diagnosis is uncommon. 3) In a second phase, IgG1 antibodies mediate antigen internalization, resulting in cytoskeleton alteration, aggregation of tau, and, ultimately, neuronal death and neurodegeneration. This pathological substrate parallel with a slowly progressive clinical course, and most patients are diagnosed in this phase. *PC* plasma cell, *BBB* blood-brain barrier, *FDG-PET* fluorodeoxyglucose positron emission tomography

Interestingly, clinical presentations mimicking neurodegenerative disorders appear to be more common in AE where the autoantibodies are predominantly of the IgG4 subclass, and in which specific human leukocyte antigen (HLA) associations have been reported. Indeed, LGI1-Abs are mainly of the IgG4 subclass [62, 67] and an association with the HLA ~ DRB1*07:01 allele has been reported in about 90% of LGI1-Ab patients [62, 106]. Importantly, the interplay between inflammation, autoantibodies and neurodegeneration is particularly relevant in IgLON5-Ab disease, where IgG4 is the predominant IgG subclass in most patients [47] and a tight association with specific HLA haplotypes (mainly HLA ~ DQB1*05:01) has been found [107]. While initially believed to be a primarily neurodegenerative disorder due to pathology reports showing a novel tauopathy [46], later studies revealed inflammatory infiltrates [108–110] and challenged this hypothesis [47]. Indeed, in a post-mortem series of 9 IgLON5-Ab disease patients, tau pathology was not detected in three with short disease duration, while two patients had a prominent antibody deposition (mostly IgG4) in the predilected area of tau deposition (brainstem, olivary nuclei, and cerebellar cortex), indicating that IgLON5-Ab deposition precedes the tauopathy [47]. At the same time, clinical studies show that CSF cell count inversely correlates with time to CSF analysis [49], while early administration of immunotherapy (within the first year after onset) and low pre-treatment neurofilament light chain (NfL) levels are associated with higher chances of treatment response [49]. Altogether, these findings suggest that IgLON5-Ab disease is a primary autoimmune disorder, with initial inflammatory mechanisms and subsequent neurodegenerative changes with tau deposits occurring in an advanced disease stage, as depicted in Fig. 3.

Regarding the direct pathogenic mechanisms of the autoantibodies, in rat neuronal cultures IgLON5-Abs cause irreversible internalization of surface IgLON5 along with cytoskeleton disruption, resulting in dystrophic neurites, axonal swelling, and/or other abnormalities reminiscent of those seen in primary neurodegenerative disorders [111, 112]. In addition, IgLON5-Ab patients’ sera can induce p-tau accumulation in human neural stem cells [113]. Besides IgG1-mediated antigen internalization [112], IgLON5-Abs, and especially IgG4, may also exert their pathogenic effect by preventing IgLON5 interaction with its binding partners [114], akin to previously mentioned mechanisms for LGI1- and CASPR2-Abs. Moreover, it has been recently shown that changes in the CSF concentration of IgG4 IgLON5-Abs is associated with disease severity and that IgG4 are synthesized intrathecally, highlighting their importance in the pathogenesis of the disease [115].

Lastly, pathogenic effects of IgLON5-Abs have been also reproduced in vivo where mice intracerebroventricularly infused with IgLON5-Abs showed p-tau deposition in the brainstem and hippocampus, along with some respiratory and motor alterations [116]. In another study, mice exposed to stereotactic or cerebroventricular transfer of IgLON5-Abs showed neuronal loss, microglial and astrocytic activation, increased expression of mRNA levels of inflammatory cytokines and behavioral changes [117]. Nonetheless, the question of how IgLON5-Abs trigger neurodegeneration remains to be fully answered, as none of the studies demonstrated that antibody-mediated structural or functional alterations of IgLON5 induce neurodegenerative changes.

### Role of seizures in inducing neurodegeneration

Seizures are a cardinal manifestation of AE and the main clinical features in some AE subtypes [118]. Temporal lobe seizures are characteristic of LGI1- and CASPR2-Ab encephalitis [10–12], while status epilepticus is a frequent occurrence in GABA_A_R- and GABA_B_R-Ab AE [34, 35]. Although few patients develop epilepsy in the long term [119], there is evidence that seizures with more subtle clinical manifestations, such as temporal lobe seizures, may persist for months after treatment and can only be detected with prolonged EEG monitoring or sleep investigations [120], highlighting possible underrecognition [121].

Recently, a bi-directional relationship between seizures and neurodegeneration has been proposed, stemming from clinical observation that patients with AD have a higher incidence and prevalence of seizures than the normal population [122], and that temporal lobe epilepsy (TLE) is often associated with cognitive impairment [123]. Indeed, evidence from animal models shows a link between increased neuronal activity and neurodegeneration. In one study, pilocarpine-induced seizures caused a change in neuronal tau phosphorylation dynamics, resulting in its increase [124]. Similarly, epileptiform activity in the hippocampus led to an increase in extracellular Aβ levels and plaque pathology [124–126], both in single-seizure models [125] or after induction of chronic hyperexcitability [124, 126].

Similar evidence in humans emerges mostly from pathological studies. The brain samples of TLE patients show upregulated amyloid precursor protein and an enhanced amyloidogenic pathway, alongside increased total and phosphorylated tau expression in the hippocampus [127]. Augmented tau pathology has also been found in patients with chronic epilepsy [128] or drug-resistant TLE [129], where it also correlated with cognitive impairment [129]. Additionally, one study found increased levels of Aβ1–42 in the CSF of epilepsy patients compared to controls [130]. On the other hand, seizures may also exacerbate cognitive impairment and pathology progression in AD, as one study found increased Aβ and tau deposits in AD patients suffering from seizures compared to those without [126]. Overall, growing data show that seizures may trigger and promote neurodegeneration, and how these mechanisms may participate in AE should be explored in future studies.

### Pre-existing pathology and individual predisposition

Recently, some experts have hypothesized that AE may act as a trigger to either initiate or exacerbate pre-existing AD-like pathology in susceptible individuals [124, 125]. This theory stems from the latest descriptions of tau pathology in IgLON5-Ab disease, as previously mentioned, and from a recent observation of AD intermediate pathology and brain positron-emission tomography (PET) tau accumulation in LGI1-Ab patients [131]. More specifically, Day et al. employed flortaucipir, a PET tau tracer, to investigate tau neuropathology in four LGI1-Ab patients, and found that two had increased deposits [131]. Moreover, brain autopsy in one of these two patients revealed both amyloid and tau accumulation, which were classified as intermediate AD neuropathological changes. The authors suggested that inflammation related to AE may increase/stimulate the accrual of neurofibrillary tau pathology in susceptible individuals.

In reality, the pathogenesis of most neurodegenerative disorders is multifaceted, involving both genetic and environmental factors, and diverse mechanisms of brain injury may facilitate the onset and spread of neurodegeneration in genetically predisposed patients. Several studies show that apolipoprotein APOε4 carriers have an increased risk of brain amyloid deposition in various conditions, ranging from normal aging [132] to diabetes [133], herpes simplex encephalitis [134] and traumatic brain injury [135]. Interestingly, a study showed that the APOε4 allele was associated with increased amyloid plaque pathology in patients with TLE, even as young as 40 years old, suggesting that seizures may trigger the development of an AD-like pathology in susceptible patients [136]. Similarly, presence of the microtubule-associated protein tau (MAPT) H1/H1 genotype has been linked to a higher predisposition to neurodegenerative diseases [137]. Importantly, IgLON5-Ab patients were found to have a significantly higher frequency of the H1/H1 genotype compared to healthy controls [138], hinting to an underlying susceptibility to develop the well-known tauopathy of IgLON5-Ab disease, possibly triggered by the inflammatory response [115].

These findings, although sparse, hint that inflammation may spark neurodegeneration in genetically predisposed individuals. Interestingly, some case reports have described the concomitant occurrence of AE and AD [131, 139] and these patients represent a rare but potentially unique paradigm to study how inflammation might trigger and/or shape neurodegenerative mechanisms in vivo.

## Role of the immune system in primarily neurodegenerative disorders

Inflammation has recently sparked global interest as a major player in the pathogenesis of neurodegenerative disorders [57]. However, as opposed to AE where dysfunction of the adaptive immune response seems to be prevalent, dysregulation of the innate immunity likely prevails in neurodegenerative disorders. For instance, AD is known to be associated with several variants of genes involved in the innate immune system and microglial function [140], such as the microglial triggering receptor expressed on myeloid cells 2 (TREM2) [141, 142]. In initial AD stages, microglia could contribute to amyloid clearance and exert a neuroprotective role, but could later acquire detrimental mechanisms which initiate and exacerbate AD pathology [143]. Indeed, chronically activated microglia produces reactive oxygen species and inflammatory molecules, leading to glial activation, neuronal dysfunction and death. In different studies, pro-inflammatory cytokines secreted by microglia exerted a harmful effect on hippocampal neurogenesis [144] and promoted Aβ accumulation, both by upregulating the production of beta-secretase [145] and by releasing apoptosis-associated speck-like proteins that act as a core for Aβ aggregation [146].

Additionally, microglia directly spread tau pathology through exosome secretion and its depletion halted tau propagation both in vitro and in vivo [147]. In addition to evidence from cellular and animal models, translocator protein (TSPO) PET studies have demonstrated microglial activation in humans with AD [148, 149], including reports that microglial activation inversely correlates with hippocampal volume and metabolism [148, 150], while correlating positively with amyloid load [148]. With a similar approach, microglial activation has been demonstrated in vivo in other neurodegenerative disorders [151], such as Parkinson’s disease [152], MSA [153], Lewy body dementia [154] and ALS [155]. Interestingly, microglial activation in the temporal lobe has been recently described in LGI1-Ab AE [156, 157], both in the acute phase but also persisting for up to 35 months after onset [156].

In addition to microglia, other glial cells seem to play a role in the pathogenesis of neurodegenerative disorders, including AD [158]. For example, astrocyte activation in AD leads to dysregulated neurotransmitter release and disruption of the blood–brain barrier, further exacerbating synaptic dysfunction and neuronal loss [159, 160].

Moreover, the association of AD with specific polymorphisms in HLA class II loci [140] could suggest that antigen presentation and thus, the adaptive immune system, may also be at play in the underlying pathogenesis. The potential contribute to AD pathogenesis by lymphocytes and other immune system cells is discussed in detail elsewhere [158].

## Conclusions and future perspectives

The growing evidence from both preclinical and clinical studies suggests a fundamental interplay between inflammation and neurodegeneration. Clinical manifestations resembling neurodegenerative disorders are not uncommon in AE, and conversely, inflammatory processes, though often overlooked, are being increasingly recognized as potential contributors to neurodegeneration.

The clinical manifestations of AE may be insidious and slowly progressive, and particularly when inflammatory MRI and/or CSF findings are absent, physicians must remain vigilant for clinical features suggestive of an autoimmune etiology, as their prompt identification and subsequent neural antibody testing are crucial for diagnosis. Unlike neurodegenerative disorders, the course of AE can be modified with appropriate immunotherapy, making early recognition essential to prevent misdiagnosis and ensure optimal treatment.

Conversely, understanding the role of neuroinflammation in neurodegenerative disease could provide novel insights into underlying disease mechanisms and potential therapeutic targets. Microglial activation in primarily neurodegenerative disorders is merely a piece in an enigmatic, multifaceted puzzle which has yet to be revealed.

While the precise genetic, molecular and cellular pathways driving neurodegeneration in AE remain to be fully elucidated, current findings highlight the need for further research into the complex interplay between autoimmunity and neurodegeneration. A deeper understanding of the role of IgG4 and specific HLA haplotypes in AE pathogenesis may yield valuable insights. Continued research into these mechanisms has the potential not only to advance our understanding of AE but also to pave the way for novel therapeutic approaches in neurodegenerative disorders.

## Data Availability

Not applicable.
